# Palatal mucosa derived fibroblasts present an adaptive behavior regarding cytokine secretion when grafted onto the gingival margin

**DOI:** 10.1186/1472-6831-14-21

**Published:** 2014-03-20

**Authors:** Fabíola Pontes Azevedo, Ana Carolina Faria Morandini, Carla Renata Sipert, Thiago José Dionísio, Carlos Ferreira Santos, Carla Andreotti Damante, Maria Lúcia Rubo de Rezende, Adriana Campos Passanezi Sant’Ana, Sebastião Luiz Aguiar Greghi

**Affiliations:** 1 Department of Prosthodontics, Bauru School of Dentistry, University of São Paulo, Bauru, São Paulo, Brazil; 2 Department of Biological Sciences, Bauru School of Dentistry, University of São Paulo, Bauru, São Paulo, Brazil

**Keywords:** Periodontitis, Gingival fibroblasts, Cytokines, Chemokine, Inflammation

## Abstract

**Background:**

Considering that grafted gingival tissue might have to be adapted to the receptor area and that fibroblasts have the ability to respond to bacterial stimuli through the release of various cytokines, this study investigated whether fibroblasts from the palatal mucosa behave differently when grafted onto the gingival margin regarding cytokine secretion.

**Methods:**

Biopsies from the palatal mucosa were collected at the time of free gingival graft surgery, and after four months re-collection was performed upon surgery for root coverage. Fibroblasts were isolated by the explant technique, cultured and stimulated with *Porphyromonas gingivalis* (*Pg*) and *Escherichia coli* (*Ec*) LPS for 24 or 48 h for comparative evaluation of the secretion of cytokines and chemokines, such as IL-6, IL-8/CXCL8, MIP-1α/CCL3, TGF-β, VEGF and CXCL16. Unstimulated cells were used as the control group. Cells were tested for viability through MTT assay, and secretion of cytokines and chemokines was evaluated in the cell supernatants by Enzyme-Linked Immunosorbent Assay (ELISA).

**Results:**

Fibroblasts from the palatal mucosa maintained the same secretion pattern of IL-6 when grafted onto the gingival margin. On the contrary, fibroblasts from the marginal gingival graft showed increased secretion of IL-8/CXCL8 even in the absence of stimulation. Interestingly, MIP-1α/CCL3 secretion by fibroblasts from the marginal gingival graft was significantly increased after 48 hours of stimulation with *Pg* LPS and after 24 h with *Ec* LPS. Only fibroblasts from the marginal gingival graft showed secretion of TGF-β. VEGF and CXCL16 secretion were not detected by both subsets of fibroblasts.

**Conclusion:**

Fibroblasts from the palatal mucosa seem to be adapted to local conditions of the site microenvironment when grafted onto the gingival marginal area. This evidence supports the effective participation of fibroblasts in the homeostasis of the marginal periodontium through secretion modulation of important inflammatory mediators.

## Background

Gingival tissue exposure to bacterial plaque can result in tissue inflammation with clinical signs of color change, size, shape, consistency and bleeding with the possibility of alveolar bone loss due to periodontal disease progression [1]. The cumulative effect and repetitive pathological events to gingival tissue can lead to the occurrence and progression of gingival recession, especially in cases with narrow band or absence of attached gingiva [2-4].

According to the American Academy of Periodontology, periodontal plastic surgery may be indicated to increase the gingival width, creating a deeper vestibule in sites with abnormal frenum attachment and inadequate attached gingiva [5]. Even though the keratinized mucosa width and gingiva are genetically determined, they can be affected by the presence of bacterial plaque associated with inflammation or by mechanical interventions [6].

It has been shown that fibroblasts, besides being the most important cells in the maintenance and remodeling of connective tissue extracellular matrix, are involved in immune and inflammatory host response in periodontal disease, as they are the predominant structural cells found in periodontal infections by anaerobic bacteria such as *Porphyromonas gingivalis (Pg)* and can be stimulated to release inflammatory cytokines upon *Pg* and its subproducts challenge [7-11]. Besides being sensitive to their own cytokines and growth factors, they can directly interact with bacteria and their virulence factors, including lipopolysaccharide (LPS) in periodontal lesions [12].

Regarding gingival fibroblasts role in response to *Pg* LPS through cytokines release, it was previously demonstrated that these cells show differential behavior from periodontal ligament from the same donors, confirming the individuality of the anatomical region in determining the cell response [9]. Specifically, for IL-6 and IL-8/CXCL8, a well-known chemokine required for neutrophils recruitment, it was reported that these cells are able to secrete huge amounts when stimulated by *Pg* LPS [8,13-15]. Additionally, gingival fibroblasts also secrete the chemokine MIP-1α/CCL3 implying on monocytes chemoattraction in periodontal disease [16] as well as TGF-β that induces the expression of pro-collagen type I [17]. VEGF is another cytokine related to the etiology of periodontal disease due to the ability to increase vascular permeability that contributes to the dissemination of inflammation, allowing inflammatory mediator release by cells in the inflamed tissue and enabling the formation of new blood vessels [18]. Also, the chemokine CXCL16 is induced by other cytokines promoting gingival fibroblasts proliferation and leukocyte migration into inflamed tissues helping the periodontal tissues reshuffle [19,20]. To the best of our knowledge, no previous work has addressed the secretion profile of the aforementioned cytokines comparing the fibroblasts from palatal mucosa (usually used as an autogenous donor site for gingival graft procedures) with cells originated from marginal gingival area.

Grafted gingival tissue might have to be adapted to the receptor area and there is a large body of evidence that gingival fibroblasts are able to react to several stimuli through cytokines and chemokines release, which in turn play an important role in inflammatory response. Therefore, this study aimed to investigate whether the secretion of cytokines and chemokines including repair mediators by human gingival fibroblasts would be modulated when grafted from the palatal mucosa **on**to the gingival marginal area in a free gingival graft procedure.

## Methods

### Sample collection and fibroblasts primary culture

After approval by the Institutional Review Board of the Bauru School of Dentistry, University of São Paulo, #055/2011, three systemically and periodontally healthy individuals (28, 38 and 50 years-old, 3 females) were selected in Periodontics Clinics, all of whom needed root coverage in areas with scarce keratinized mucosa. They had no signs of gingival inflammation, no bleeding on probing or critical probing depth. All individuals were submitted to anamnesis, periodontal clinical examination and radiographic exams. The inclusion criteria were individuals with deficient keratinized mucosa, both in quantity and quality, without systemic complications that could contraindicate the surgical procedures, and that provided written informed consent to participate in the study.

The palatal mucosa specimens, sized 3x3 mm, were immediately obtained when an epithelium-connective tissue graft was removed from the palatal donor site on the premolars area during free gingival graft surgery [21]. After four months, specimens from gingival graft were obtained from the marginal area, during a second surgical procedure for root coverage. Fibroblasts were isolated by the explant technique as previously described [8-11]. Briefly, the specimens were processed immediately in Dulbecco's modified Eagle's medium (DMEM; Invitrogen Life Technologies, Carlsbad, CA, USA) supplemented with 20% fetal bovine serum (FBS; Gibco, Invitrogen Life Technologies, Carlsbad, CA, USA). The epithelial layer was removed and the specimens were minced as small as possible. Tissue fragments of palatal mucosa or gingival graft were plated separately in Petri dishes and covered with DMEM supplemented with 20% FBS and antibiotics (600 μL/mL penicillin, 300 μL/mL gentamicin sulfate and 100 μL/mL amphotericin B). The explants were placed in 25 cm^2^ flasks and incubated at 37°C in a humidified atmosphere of 5% CO_2_. The medium (DMEM 10% FBS) was changed every 2–3 days and cultures were maintained until fibroblasts reached confluence. After confluence, the fibroblasts were harvested and used between the fourth and eighth passages [8-11].

### Fibroblasts stimulation

The cells were seeded in 24 well plates at a density of 2×10^4^ cells/well and incubated at 37°C in a humidified atmosphere of 5% CO_2_ during 18 hours in DMEM 1% FBS. After overnight adhesion, DMEM containing 1 μg/mL of *Pg* LPS (Invivogen, San Diego, CA, USA) or *Ec* LPS (Invivogen) was added to the wells for 24 or 48 h. Non-stimulated cells were used as controls. Both *Pg* LPS and *Ec* LPS were ultrapure and purchased from Invivogen.

### Phenotypic characterization of fibroblasts

Cells cultured from the palatal mucosa and gingival graft were characterized as fibroblasts by their morphology and staining with fibroblast surface protein (FSP; ab 11333, Abcam, Cambridge, UK) by means of immunostaining [11]. Cells were seeded in an 8-well plate with 1×10^4^ cells per well and incubated at 37°C with 5% CO_2_ during 18 hours to adhere in subconfluence. Subsequently, the wells were divided into groups (control, *Pg* LPS, *Ec* LPS and negative control, which was performed without the primary antibody) in duplicate, to cell stimulation. Cells were fixed with paraformaldehyde 4% (15 min) and were washed 3 times with 1x PBS and incubated with PBS BSA 3% (30 min at room temperature) followed by primary monoclonal antibody incubation to fibroblast surface marker diluted at 1:100 (2 μg/mL final concentration), and finally with fluorescein-conjugated secondary antibody (1:400) (Abcam) at 37°C in the dark for 1 hour. Coverslips were mounted with mounting medium VECTASHIELD Hard-Set Mounting Medium containing 49,6-diamidino-2-Phenylindole (DAPI; H-1500, Vector Laboratories, Burlingame, CA, USA) and analyzed by confocal microscope laser scanning (TCS model, SPE, Leica Mannheim, Germany).

### Cell viability

Cell viability was analyzed from enzymatic activity with a colorimetric assay MTT [3-(4,5-Dimethylthiazol-2-yl)-2,5-diphenyltetrazolium bromide]. Briefly, cells were washed with 1× PBS to remove culture medium and MTT (5 mg/mL) was added to the groups (control, *Pg* LPS and *Ec* LPS) or cell-free wells (blank). The plate was incubated during 4 hours at 37°C with 5% CO_2_. After incubation, the plate was centrifuged (200 *g* during 7 minutes at 21°C) and MTT solution was removed to add dimethylsulfoxide (DMSO; Sigma-Aldrich, St. Louis, MO). The optical density of the wells was determined using a plate reader (Fluostar Optima, BMG Labtech, Ortenberg, Germany) in the wavelength of 570 nm.

### Enzyme-linked immunosorbent assay (ELISA)

ELISA was performed following the manufacturer’s instructions to determine the protein levels of IL-6 (R&D System, Minneapolis, MN, EUA), IL-8/CXCL8 (R&D System, Minneapolis, MN, EUA), MIP-1α/CCL3 (R&D System, Minneapolis, MN, EUA), TGF-β (eBioscience, San Diego, CA, USA), VEGF (PeproTech, London, UK) and CXCL16 (PeproTech, London, UK). Briefly, plates of 96 wells were incubated with capture antibody anti-IL-6, IL-8/CXCL8, MIP-1α/CCL3, TGF-β, VEGF or CXCL16 diluted in 1x PBS buffer. After blocking for 1 hour to avoid non-specific binding, 100 μl of standard IL-6, IL-8/CXCL8, MIP-1α/CCL3, TGF-β, VEGF and CXCL16 and culture supernatants were placed. The cytokines were detected by horseradish peroxidase-labeled monoclonal antibody to each target after addition of 100 μl anti-human IL-6, IL-8/CXCL8, MIP-1α/CCL3, TGF-β, VEGF and CXCL16 biotinylated antibodies were placed in each well and incubated for 2 hours at room temperature. The microplate was washed to remove unbound enzyme-labeled antibodies. The amount of horseradish peroxidase bound to each well was determined by the addition of 100 μl substrate solution. The reaction was stopped by the addition of 100 μl of 1 M sulfuric acid, and the plates were read at 450 nm (Synergy™ MX Monochromator Based Multi-Mode Microplate Reader, BioTek Instruments, Inc, Winooski, VT, USA). The concentrations of IL-6, IL-8/CXCL8, MIP-1α/CCL3, TGF-β, VEGF and CXCL16 were determined by interpolation from a standard curve and presented as pg/mL for duplicate assays of duplicate samples of each of the tested conditions.

### Statistical analysis

Comparisons for viability through MTT among samples were performed using One-way ANOVA test followed by Tukey test. ELISA data normality was verified by the Kolmogorov-Smirnov method. The statistical differences were determined by three-way ANOVA followed by Tukey test. Statistical analyses were performed with STATISTICA (version 11.0; StatSoft Inc). P values < 0.05 were considered significant. The results were expressed as mean and one standard deviation of the mean.

## Results

### Phenotypic characterization of fibroblasts

**Figure 1:**
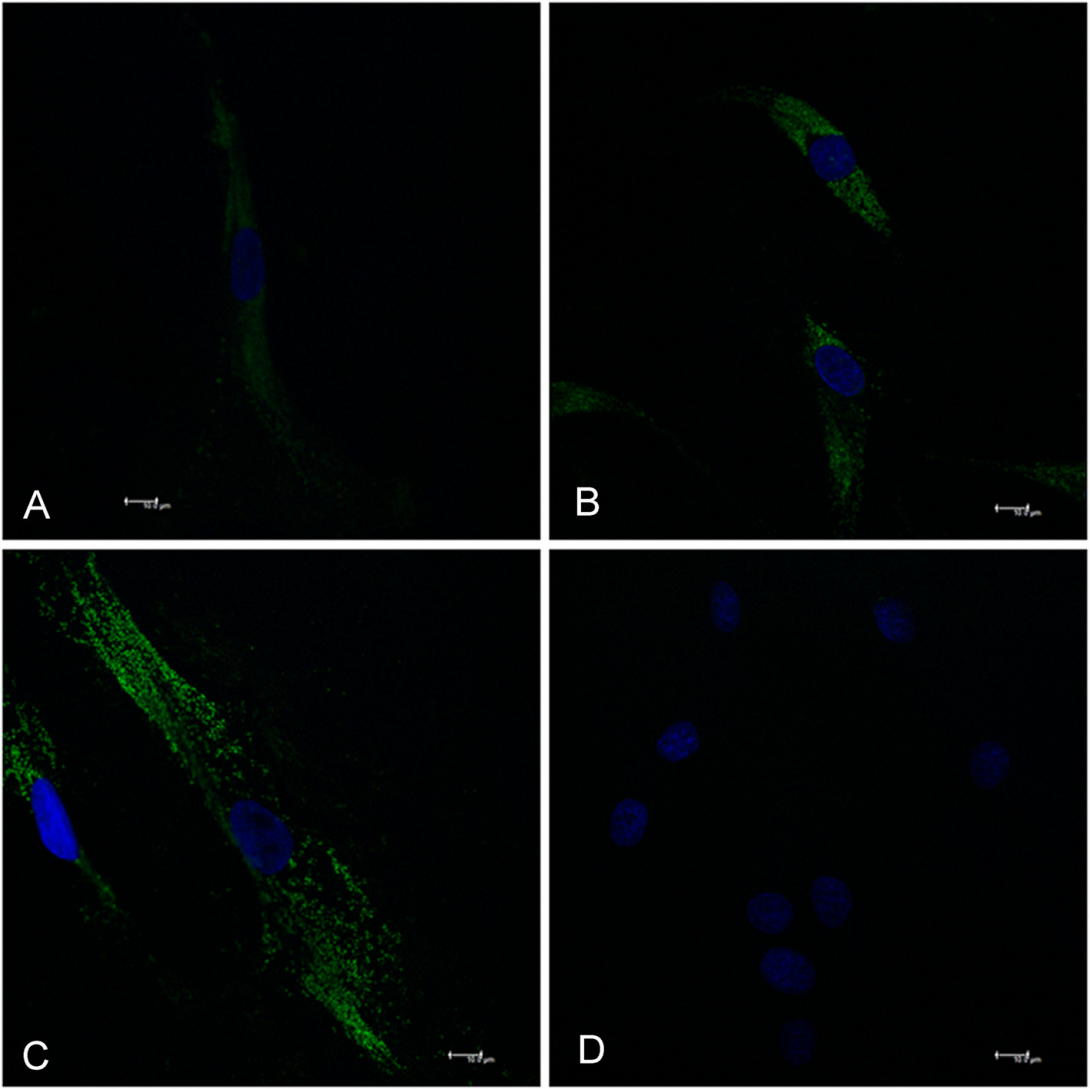
**Cells characterization by fibroblast protein surface staining.** Cultured cells were immunostained for fibroblast surface protein (FSP) and analyzed by a confocal microscope at an increase of 63x. **(A)** control; **(B)** stimulated by *Ec* LPS; **(C)** stimulated by *Pg* LPS and **(D)** negative control, labeled nuclei in blue (DAPI).

Cellular staining with a fibroblast surface marker (FSP) was performed to confirm the fibroblastic phenotype. Fibroblasts were analyzed by immunofluorescence in all groups (control, *Pg* LPS, *Ec* LPS and negative control). Positive staining was observed for all groups (Figure 1), with *Pg* LPS (Figure 1C) and *Ec* LPS (Figure 1B) groups being slightly higher. For the negative control group (Figure 1D), which did not receive primary antibody, no signal was detected. This result confirms the characterization of the cultured cells as fibroblasts.

### Cell viability

**Figure 2:**
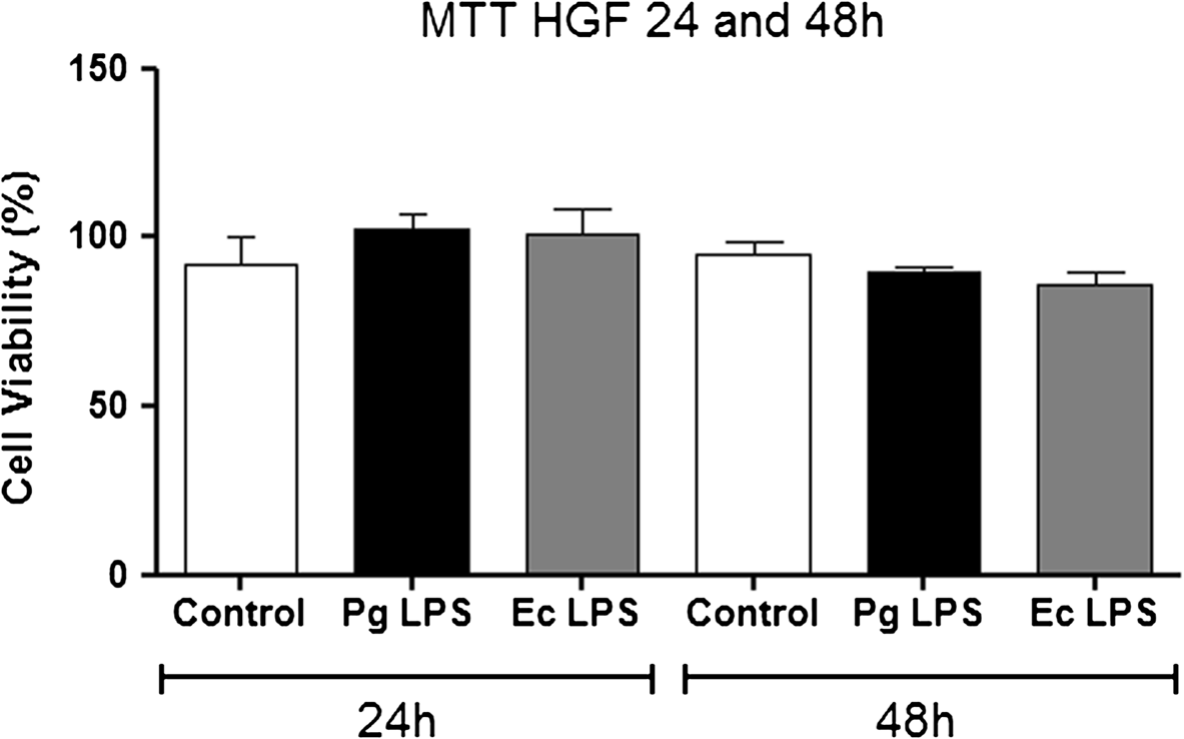
**Cells viability.** Fibroblasts viability challenged with *Pg* LPS and *Ec* LPS evaluated at 24 and 48 hours in the control (non-stimulated) and test groups (stimulated with *Pg* LPS and *Ec* LPS) through MTT assay.

Cell viability was evaluated after 24 and 48 h, in the control (non-stimulated) and testing groups (stimulated with *Pg* LPS and *Ec* LPS). There was no difference in cells viability with the stimuli used after 24 and 48 h (Figure 2).

### Cytokines secretion comparing fibroblasts from the palatal mucosa with the marginal gingival grafted cells

**Figure 3:**
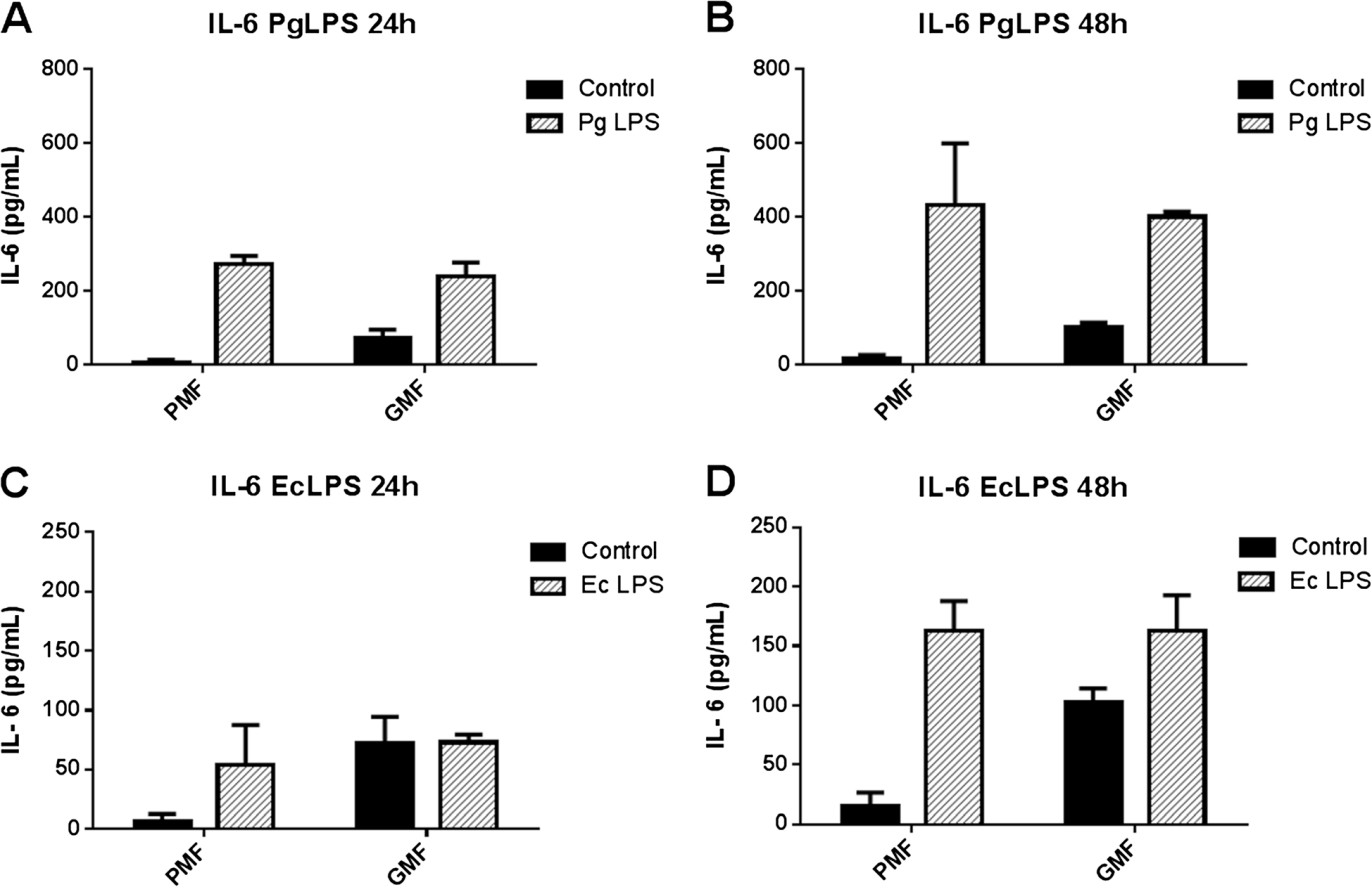
**IL-6 secretion by human fibroblasts cultured from palatal mucosa and marginal gingival graft. A-D**: primary cell cultures were obtained from periodontal tissues from three healthy subjects and, after the fourth passage, were stimulated with LPS from *P. gingivalis* and from *E. coli* in a concentration of 1 μg/mL. Culture medium without stimuli was used as the control. ELISA was performed for the quantification of the cytokines on the cell culture supernatants. Representative figure of the average of three independent experiments, n = 3. *P < 0.05 was considered significantly different. PMF = palatine mucosa fibroblasts. GMF = marginal gingival fibroblasts.

When the targets of this study were compared between the two subpopulations of fibroblasts, IL-6 secretion was statistically significant after 24 and 48 h of *Pg* LPS stimulation (Figure 3A and B) by both subtypes of fibroblasts (from the palatal mucosa and gingival graft in marginal area), and the difference was greater with 48 h (Figure 3B). On the other hand, the secretion after *Ec* LPS stimulation was only statistically significant after 48 h, by both subtypes of fibroblasts (Figure 3D). Additionally, IL-6 secretion was greater by non-stimulated fibroblasts from the marginal gingival graft (Figure 3).

**Figure 4:**
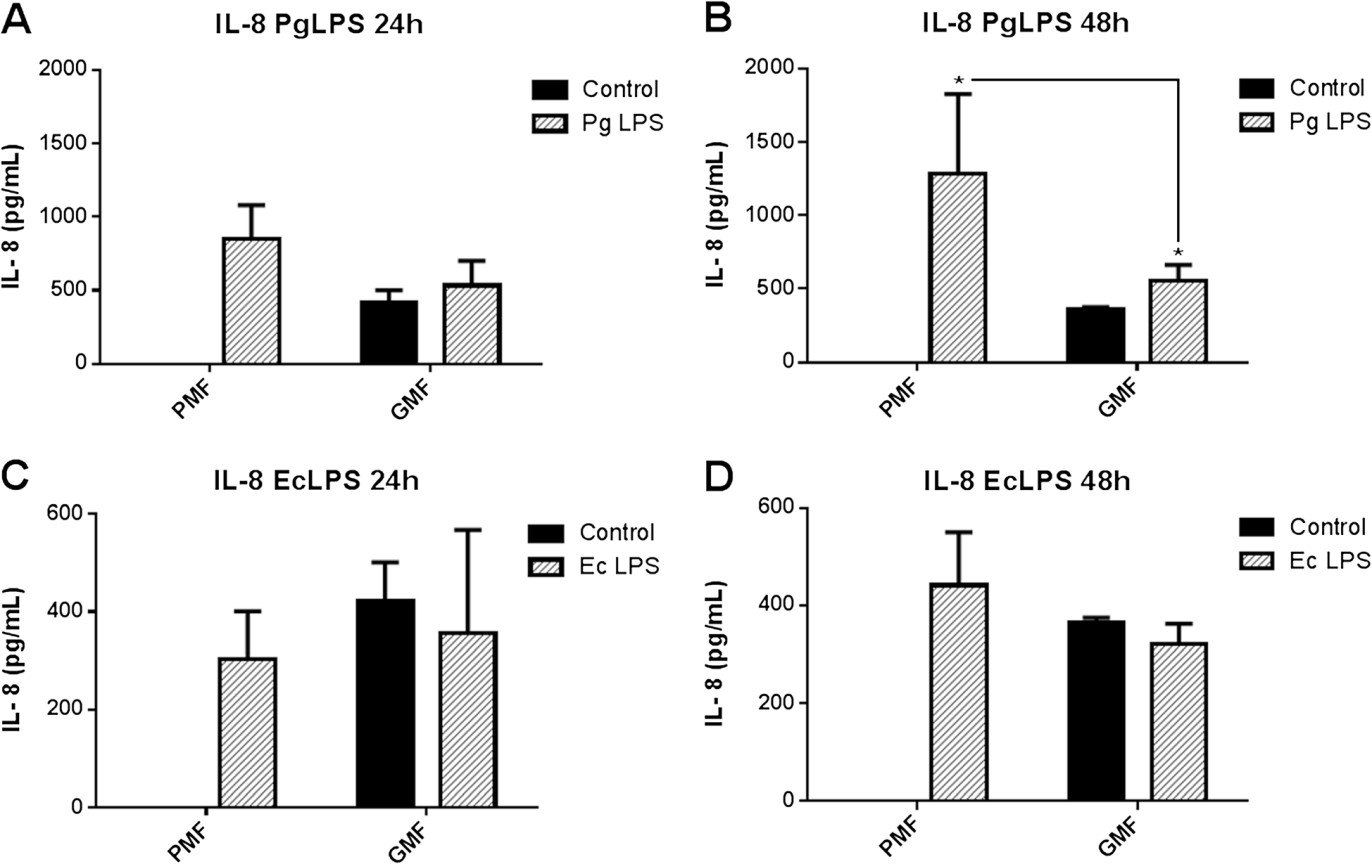
**IL-8/CXCL8 secretion by human fibroblasts cultured from palatal mucosa and marginal gingival graft. A-D**: primary cell cultures were obtained from periodontal tissues from three healthy subjects and, after the fourth passage, were stimulated with LPS from *P. gingivalis* and from *E. coli* in a concentration of 1 μg/mL. Culture medium without stimuli was used as the control. ELISA was performed for the quantification of the cytokines on the cell culture supernatants. Representative figure of the average of three independent experiments, n = 3. *P < 0.05 was considered significantly different. PMF = palatine mucosa fibroblasts. GMF = marginal gingival fibroblasts.

Fibroblasts from the palatal mucosa secreted more IL-8/CXCL8 than fibroblasts from the marginal gingival graft when stimulated by *Pg* LPS with a statistically significant difference at 48 h (Figure 4A and B). Also, regarding this chemokine, fibroblasts from the palatal mucosa showed an increase at both 24 h and 48 h when stimulated by *Ec* LPS (Figure 4C and D), which was not observed by cells from the marginal gingival graft.

**Figure 5:**
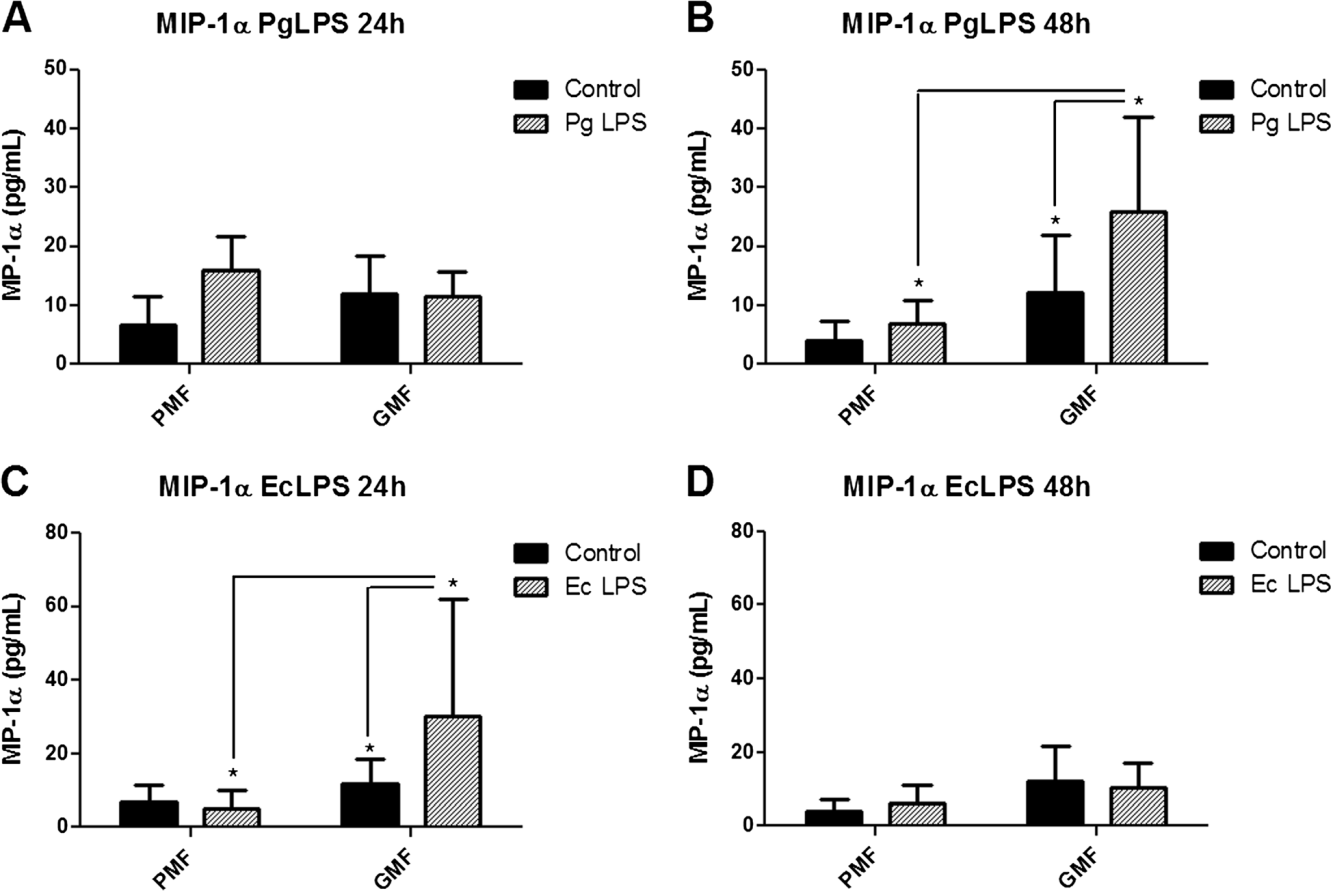
**MIP-1α/CCL3 secretion by human fibroblasts cultured from palatal mucosa and marginal gingival graft. A-D**: primary cell cultures were obtained from periodontal tissues from three healthy subjects and, after the fourth passage, were stimulated with LPS from *P. gingivalis* and from *E. coli* in a concentration of 1 μg/mL. Culture medium without stimuli was used as the control. ELISA was performed for the quantification of the cytokines on the cell culture supernatants. Representative figure of the average of three independent experiments, n = 3. *P < 0.05 was considered significantly different. PMF = palatine mucosa fibroblasts. GMF = marginal gingival fibroblasts.

In this study, MIP-1α/CCL3 was observed to be constitutively secreted by both fibroblasts subtypes (Figure 5). Moreover, there was no statistically significant difference in MIP-1α/CCL3 secretion between unstimulated fibroblasts (control group) and fibroblasts stimulated with *Pg* or *Ec* LPS from the palatal mucosa (Figure 5). However, statistically significant difference was observed in MIP-1α/CCL3 secretion after 48 h when fibroblasts from the marginal gingival graft were stimulated with *Pg* LPS (Figure 5B) and after 24 h when challenged by *Ec* LPS (Figure 5C).

**Figure 6:**
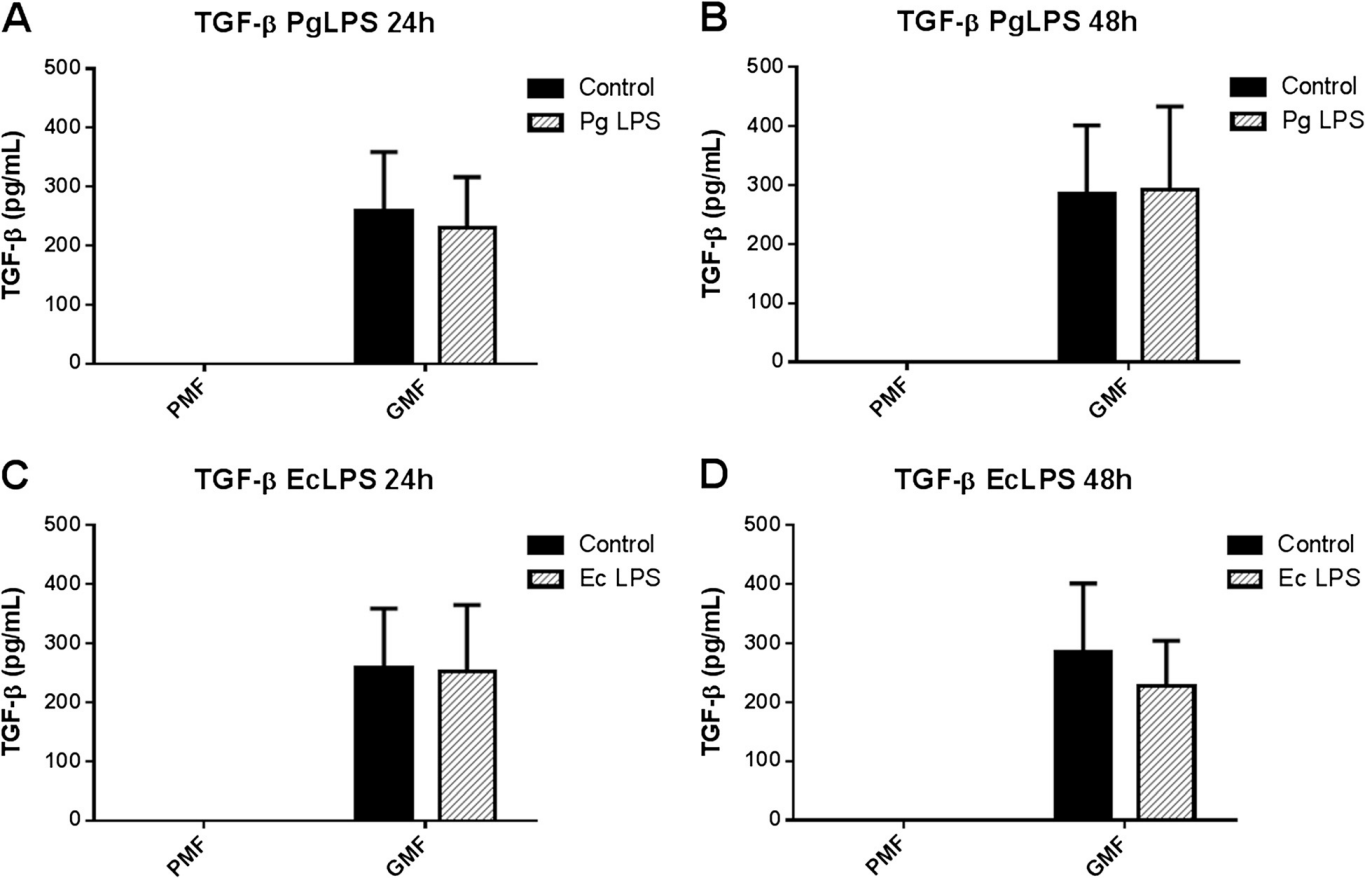
**TGF-β secretion by human fibroblasts cultured from palatal mucosa and marginal gingival graft. A-D**: primary cell cultures were obtained from periodontal tissues from three healthy subjects and, after the fourth passage, were stimulated with LPS from *P. gingivalis* and from *E. coli* in a concentration of 1 μg/mL. Culture medium without stimuli was used as the control. ELISA was performed for the quantification of the cytokines on the cell culture supernatants. Representative figure of the average of three independent experiments, n = 3. *P < 0.05 was considered significantly different. PMF = palatine mucosa fibroblasts. GMF = marginal gingival fibroblasts.

When TGF-β secretion was evaluated, fibroblasts from the palatal mucosa showed no detectable levels even in the presence of *Pg* LPS (Figure 6A and B) or *Ec* LPS (Figure 6C and D). On the contrary, fibroblasts from the marginal gingival graft showed a constitutive TGF-β secretion and there was no statistically significant difference between stimulated and unstimulated cells, both at 24 h and 48 h (Figure 6). VEGF and CXCL16 secretion was not detected either on fibroblasts supernatant cultured from the palatal mucosa neither from the marginal gingival tissue, regardless stimulation by *Pg* or *Ec* LPS.

## Discussion

Fibroblasts are not a unique cell group across the different anatomical regions [9,22-24]. They are the predominant cells in periodontal connective tissue [12], and are involved in the host immune response by releasing inflammatory cytokines [7] and repair mediators [25,26]. To the best of the author’s knowledge, this is the first study to investigate and compare the profile of mediators secretion by human fibroblasts cultured from non-marginal palatal mucosa with the marginal gingival grafted tissue, indicating the differences or similarities between these cells in contributing for cytokines secretion upon bacterial LPS stimulation.

In the present study, it was observed that both fibroblasts derived from palatal mucosa and marginal gingival graft secreted IL-6 cytokine similarly when stimulated by *Pg* or *Ec* LPS. We recognize that it would have been valuable to investigate mRNA expression in order to better understand whether transcript levels would be different between them. This could give us some information about possible post transcriptional modifications that can alter production of cytokines. Previous studies [8,14,15,27,28] have shown that fibroblasts exposed to bacterial stimuli such as LPS showed significant IL-6 expression, indicating the importance of this cytokine in the host immune and inflammatory response in periodontitis, but the present investigation also revealed increased IL-6 secretion at 48 h for both subtypes of fibroblasts. Here, there was IL-6 constitutive secretion by unstimulated cells from the palatal mucosa and also from the marginal gingival graft, being statistically significant for the latter. Scheres et al. [14] showed that non-stimulated gingival fibroblasts expressed and secreted more IL-6 than periodontal ligament fibroblasts. This suggested that gingival fibroblasts have a higher state of activity because they are more susceptible to have contact with periodontal pathogens and here we highlight this important role for both subtypes of gingival cells.

It is shown here that fibroblasts from the palatal mucosa only secreted IL-8/CXCL8 chemokine when stimulated with bacterial LPS. When these cells were grafted onto the gingival marginal area, after 4 months IL-8/CXCL8 chemokine secretion was observed even in the absence of LPS stimuli. This change in the expression profile may be related to the closer contact of the cells with bacterial virulence factors, such as *Pg* LPS, on the marginal periodontium. Previous studies [9,13-15,29] have shown that fibroblasts stimulated by *Pg* LPS are able to produce IL-8, and fibroblasts from different anatomical regions show heterogeneity on chemokine profile secretion [14]. Therefore, it further emphasizes that the heterogeneous secretion of IL-8/CXCL8 in the current study may have occurred by the environmental influence on the secretion of fibroblast mediators grafted onto a new anatomical region, in which there is adaptation and proliferation of these cells.

When the chemokine MIP-1α/CCL3 was investigated, it was observed that when fibroblasts from the palatal mucosa were grafted onto the marginal gingival area, they maintained similar cytokine constitutive secretion in the absence of bacterial stimulation. However, fibroblasts from the marginal gingival graft showed a higher secretion compared with fibroblasts from the palatal mucosa only after 48 h when stimulated by *Pg* LPS, showing a time-dependent expression. Again, the increased chemokine secretion, in this case MIP-1α/CCL3, by fibroblasts from the marginal gingival graft might have occurred due to prior stimulation in the marginal area by dental plaque in the sulcus, suggesting that these cells present an adaptive behavior when grafted onto the gingival margin. A previous work by our group [9] showed that gingival and periodontal ligament fibroblasts behaved differently on MIP-1α/CCL3 secretion when stimulated by *Pg* LPS, and also showed an increased MIP-1α/CCL3 secretion when cells were challenged by *Pg* LPS in low concentrations such as 0.1 μg/mL, which increased over time.

Concerning TGF-β secretion, fibroblasts cultured from the palatal mucosa and from the marginal gingival graft showed a heterogeneous behavior. Fibroblasts from the palatal mucosa did not show TGF-β secretion even against the bacterial stimuli applied. On the contrary, fibroblasts from the marginal gingival graft secreted TGF-β, without any difference between stimulated and non-stimulated cells. One study [30] showed that fibroblasts from periodontal ligament expressed TGF-β but its production was not affected by bacterial LPS, suggesting that TGF-β accumulate in inflammatory conditions suppressing the cellular immune function, yet without leading to tissue destruction through stimulation with bacterial LPS. Another study comparing periodontal ligament and gingival cells [8] found that TGF-β secretion depended on the stimulus concentration, mainly for gingival fibroblasts, being higher when challenged with 10 μg/mL of LPS, whereas the concentration of 1 μg/mL as used in the current study, showed no significant differences compared with the non-stimulated fibroblasts. The increased TGF-β secretion found here by marginal grafted cells could be related to tissue repair and might have occurred by the continuous repair need, since the constant presence of dental sulcus microbiota induces marginal changes.

Lastly, the results indicated that neither fibroblasts from the palatal mucosa nor from the marginal gingival graft secreted VEGF nor CXCL16, according to the stimuli used. Previous studies [31,32] have shown that human gingival fibroblasts stimulated by LPS, vesicle and proteins from *Pg* and *Aa* extracellular membrane were able to produce VEGF when stimulated by such agents. Maybe in this study the time and concentration of LPS used were not sufficient for the induction of VEGF. Regarding CXCL16, it was reported that this expression was induced by other cytokines such as IFN-γ and TNF-α, and their secretion was mediated by ADAM 10 membrane receptor, which regulates CXCL16 function on inflamed tissues [33]. Another work [19] showed that CXCL16 expression by gingival fibroblasts was controlled by several cytokines such as IL-1β, TNF-α and IFN- γ. In fact, one could speculate that CXCL16 secretion was not detected in the current study because fibroblasts were stimulated only by bacterial LPS, and the action of other cytokines or other stimuli might be necessary for secretion of CXCL16 by gingival fibroblasts [19,33].

The heterogeneous secretion of inflammatory and repair mediators may indicate that fibroblasts grafted onto a new anatomical region (gingival margin) may be influenced by the microenvironment at the moment these cells are in contact with bacterial products, participating more effectively in the context of the inflammatory response.

## Conclusions

Fibroblasts from the palatal mucosa present an adaptive behavior regarding cytokines secretion when grafted onto the gingival margin, showing difference in the secretion of important inflammatory mediators to the marginal periodontium homeostasis.

## Abbreviations

Pg: *Porphyromonas gingivalis*; Ec: *Escherichia coli*; ELISA: Enzyme-linked immunosorbent assay; LPS: Lipopolysaccharide; DMEM: Dulbecco's modified Eagle's medium; FBS: Fetal bovine serum; FSP: Fibroblast surface protein; BSA: Bovine serum albumin; DAPI: 49,6-diamidino-2-Phenylindole; DMSO: Dimethylsulfoxide; MTT: [3-(4,5-Dimethylthiazol-2-yl)-2,5-diphenyltetrazolium bromide.

## Competing interests

The authors declare that they have no competing interests.

## Authors’ contributions

FPA, ACFM, CRS, CFS and SLAG conceived and planned the project. FPA, ACFM, TJD and CFS designed the study, conducted laboratory experimentations, performed data analyses, conducted the statistical analysis and wrote the manuscript. FPA made surgical procedures and data collection. CAD, MLRR, ACPS, CRS and SLAG contributed to the manuscript corrections. All authors read and approved the final manuscript.

## Pre-publication history

The pre-publication history for this paper can be accessed here:

http://www.biomedcentral.com/1472-6831/14/21/prepub

## References

[B1] Mariotti A Dental plaque-induced gingival diseases Ann Periodontol 1999 4 7 19 10.1902/annals.1999.4.1.7 10863371

[B2] Löe H Anerud A Boysen H The natural history of periodontal disease in man: prevalence, severity, and extent of gingival recession J Periodontol 1992 63 489 495 10.1902/jop.1992.63.6.489 1625148

[B3] Kassab MM Cohen RE The etiology and prevalence of gingival recession J Am Dent Assoc 2003 134 220 225 10.14219/jada.archive.2003.0137 12636127

[B4] Toker H Ozdemir H Gingival recession: epidemiology and risk indicators in a university dental hospital in Turkey Int J Dent Hyg 2009 7 115 120 10.1111/j.1601-5037.2008.00348.x 19413547

[B5] Greenwell H Fiorellini J Giannobile W Offenbacher S Salkin L Townsend C Sheridan P Genco R Oral reconstructive and corrective considerations in periodontal therapy J Periodontol 2005 76 1588 1600 16171452 10.1902/jop.2005.76.9.1588

[B6] Marquez IC The role of keratinized tissue and attached gingiva in maintaining periodontal/peri-implant health Gen Dent 2004 52 74 79 15055675

[B7] Brunner J Scheres N El Idrissi NB Deng DM Laine ML van Winkelhoff AJ Crielaard W The capsule of *Porphyromonas gingivalis* reduces the immune response of human gingival fibroblasts BMC Microbiol 2010 10 1 11 10.1186/1471-2180-10-1 20064245 PMC2817674

[B8] Morandini AC Sipert CR Ramos-Junior ES Brozoski DT Santos CF Periodontal ligament and gingival fibroblasts participate in the production of TGF-β, interleukin (IL)-8 and IL-10 Braz Oral Res 2011 25 157 162 21537641 10.1590/s1806-83242011000200010

[B9] Morandini AC Sipert CR Gasparoto TH Greghi SL Passanezi E Rezende ML Sant'ana AP Campanelli AP Garlet GP Santos CF Differential production of macrophage inflammatory protein 1-α, stromal-derived factor-1, and IL-6 by human cultured periodontal ligament and gingival fibroblasts challenged with lipopolyssaccharide from *P. gingivalis* J Periodontol 2010 81 310 317 10.1902/jop.2009.090375 20151811

[B10] Morandini AC Souza PPC Ramos-Junior ES Costa CAS Santos CF MyD88 or TRAM knockdown regulates interleukin (IL)-6, IL-8 and CXCL12 mRNA expression in human gingival and periodontal ligament fibroblasts J Periodontol 2013 84 1353 1360 10.1902/jop.2012.120496 23136947

[B11] Morandini ACF Souza PPC Ramos-Junior ES Brozoski DT Sipert CR Souza Costa CA Santos CF Toll-like receptor 2 knockdown modulates interleukin (IL)-6 and IL-8 but not stromal derived factor-1 (SDF-1/CXCL12) in human periodontal ligament and gingival fibroblasts J Periodontol 2013 84 535 544 10.1902/jop.2012.120177 22680301

[B12] Bartold PM Walsh LJ Narayanan AS Molecular and cell biology of the gingiva Periodontol 2000 2000 24 28 35 10.1034/j.1600-0757.2000.2240103.x 11276872

[B13] Ara T Kurata K Hirai K Uchihashi T Uematsu T Imamura Y Furusawa K Kurihara S Wang PL Human gingival fibroblasts are critical in sustaining inflammation in periodontal disease J Periodontal Res 2009 44 21 27 10.1111/j.1600-0765.2007.01041.x 19515019

[B14] Scheres N Laine ML de Vries TJ Everts V van Winkelhoff AJ Gingival and periodontal ligament fibroblasts differ in their inflammatory response to viable *Porphyromonas gingivalis* J Periodontal Res 2010 45 262 270 10.1111/j.1600-0765.2009.01229.x 19778323

[B15] Herath TD Wang Y Seneviratne CJ Lu Q Darveau RP Wang CY Jin L *Porphyromonas gingivalis* lipopolysaccharide lipid A heterogeneity differentially modulates the expression of IL-6 and IL-8 in human gingival fibroblasts J Clin Periodontol 2011 38 694 701 10.1111/j.1600-051X.2011.01741.x 21752043

[B16] Garlet GP Martins W Jr Ferreira BR Milanezi CM Silva JS Patterns of chemokines and chemokine receptors expression in different forms of human periodontal disease J Periodontal Res 2003 38 210 217 10.1034/j.1600-0765.2003.02012.x 12608917

[B17] Arancibia R Oyarzún A Silva D Tobar N Martínez J Smith PC Tumor necrosis factor-α inhibits transforming growth factor-β-stimulated myofibroblastic differentiation and extracellular matrix production in human gingival fibroblasts J Periodontol 2013 84 683 693 10.1902/jop.2012.120225 22813343

[B18] Johnson RB Serio FG Dai X Vascular endothelial growth factors and progression of periodontal diseases J Periodontol 1999 70 848 852 10.1902/jop.1999.70.8.848 10476891

[B19] Hosokawa Y Hosokawa I Ozaki K Nakae H Matsuo T CXC chemokine ligand 16 in periodontal diseases: expression in diseased tissues and production by cytokine-stimulated human gingival fibroblasts Clin Exp Immunol 2007 149 146 154 10.1111/j.1365-2249.2007.03398.x 17459077 PMC1942022

[B20] Hosokawa Y Hosokawa I Ozaki K Nakae H Matsuo T Human gingival fibroblasts express functional chemokine receptor CXCR6 Clin Exp Immunol 2009 156 413 418 10.1111/j.1365-2249.2009.03915.x 19438592 PMC2691968

[B21] Sullivan HC Atkins JH Free autogenous gingival grafts. III. Utilization of grafts in the treatment of gingival recession Periodontics 1968 6 152 160 5243142

[B22] Scheres N Laine ML Sipos PM Bosch-Tijhof CJ Crielaard W de Vries TJ Everts V Periodontal ligament and gingival fibroblasts from periodontitis patients are more active in interaction with *Porphyromonas gingivalis* J Periodontal Res 2011 46 407 416 10.1111/j.1600-0765.2011.01353.x 21332474

[B23] Hosokawa Y Hosokawa I Ozaki K Nakae H Murakami K Miyake Y Matsuo T CXCL12 and CXCR4 expression by human gingival fibroblasts in periodontal disease Clin Exp Immunol 2005 141 467 474 10.1111/j.1365-2249.2005.02852.x 16045736 PMC1809465

[B24] Uehara A Takada H Functional TLRs and NODs in human gingival fibroblasts J Dent Res 2007 86 249 254 10.1177/154405910708600310 17314257

[B25] Nishi H Ohta K Takechi M Yoneda S Hiraoka M Kamata N Wound healing effects of gingival fibroblasts cultured in animal-free medium Oral Dis 2010 16 438 444 10.1111/j.1601-0825.2010.01654.x 20233319

[B26] Agis H Watzek G Gruber R Prolyl hydroxylase inhibitors increase the production of vascular endothelial growth factor by periodontal fibroblasts J Periodontol Res 2012 47 165 173 10.1111/j.1600-0765.2011.01415.x 21954882

[B27] Koka S Reinhardt RA Periodontal pathogen-related stimulation indicates unique phenotype of primary cultured human fibroblasts from gingiva and periodontal ligament: implications for oral health disease J Prosthet Dent 1997 77 191 196 10.1016/S0022-3913(97)70234-8 9051608

[B28] Ekhlassi S Scruggs LY Garza T Montufar-Scolis D Moretti AJ Klein JR *Porphyromonas gingivalis* lipopolysaccharide induces tumor necrosis factor-α and interleukin-6 (IL-6) secretion and CCL2 gene expression in mouse primary gingival cell lines: IL-6-driven activation of CCL2 J Periodontal Res 2008 43 431 439 10.1111/j.1600-0765.2008.01090.x 18942191 PMC2583119

[B29] Tamura M Tokuda M Nagaoka S Takada H Lipopolysaccharides of Bacteroides intermedius (Prevotella intermedia) and Bacteroides (Porphyromonas) gingivalis induce interleukin-8 gene expression in human gingival fibroblast cultures Infect Immun 1992 60 4932 4937 1328062 10.1128/iai.60.11.4932-4937.1992 PMC258250

[B30] Yamaji Y Kubota T Sasaguri K Sato S Suzuki Y Kumada H Umemoto T Inflammatory cytokine gene expression in human periodontal ligament fibroblasts stimulated with bacterial lipopolysaccharides Infect Immun 1995 63 3576 3581 7642293 10.1128/iai.63.9.3576-3581.1995 PMC173496

[B31] Suthin K Matsushita K Machigashira M Tatsuyama S Imamura T Torii M Izumi Y Enhanced expression of vascular endothelial growth factor by periodontal pathogens in gingival fibroblasts J Periodontal Res 2003 38 90 96 10.1034/j.1600-0765.2003.01646.x 12558942

[B32] Núñez MJ Novío S Balboa J Seoane J Suárez JA Freire-Garabal M Effects of resveratrol on expression of vascular endothelial growth factor in human gingival fibroblasts stimulated by periodontal pathogens Acta Odontol Scand 2010 68 239 247 10.3109/00016357.2010.494269 20507262

[B33] Abel S Hundhausen C Mentlein R Schulte A Berkhout TA Broadway N Hartmann D Sedlacek R Dietrich S Muetze B Schuster B Kallen KJ Saftig P Rose-John S Ludwig A The transmembrane CXC-chemokine ligand 16 is induced by IFN-gamma and TNF-alpha and shed by the activity of the disintegrin-like metalloproteinase ADAM10 J Immunol 2004 172 6362 6372 15128827 10.4049/jimmunol.172.10.6362

